# Statement of Principles for Health Care Journalists

**DOI:** 10.1371/journal.pmed.0020084

**Published:** 2005-03-29

**Authors:** Gary Schwitzer

**Affiliations:** University of Minnesota School of Journalism and Mass CommunicationMinneapolis, MinnesotaUnited States of America

In “The Commercialisation of Medical and Scientific Reporting” [1], Caulfield calls on journalists to ask researchers about the nature of their funding and the financial relationship of the researchers to the sponsor. This is just one principle addressed in a much broader “Statement of Principles” I wrote this past year for the Association of Health Care Journalists (http://www.ahcj.umn.edu). The statement is available online [2].
